# Veiled Truths: Iranian Women and Risky Sexual Behavior in the Context of Substance Use

**Published:** 2018

**Authors:** Effatf Merghati Khoei, Mansoureh Jamshidimanesh, Mohammad Hassan Emamian, Fatemeh Sheikhan, Kate Dolan, Kathleen T. Brady

**Affiliations:** 1- Iranian National Center for Addiction Studies (INCAS), Institution of Risk Behavior Reduction, Tehran University of Medical Sciences, Tehran, Iran; 2- School of Nursing and Midwifery, Iran University of Medical Sciences, Tehran, Iran; 3- Center for Health Related Social and Behavioral Sciences Research, Shahroud University of Medical Sciences, Shahroud, Iran; 4- Department of Midwifery, Khalkhal Branch, Islamic Azad University, Khalkhal, Iran; 5- National Drug and Alcohol Research Centre, University of NSW, Sydney, Australia; 6- Department of Psychiatry and Behavioral Sciences, The Medical University of South Carolina (MUSC), South Carolina, USA

**Keywords:** Drug abuse, Ethnography, Iranian women, Qualitative inquiry, Sexual risk behaviors

## Abstract

**Background:**

Substance use disorders and risky sexual behavior coexist for some women. Explanatory models of women’s sexuality in the context of substance use are under study. This study aimed to explore how women’s sexual behavior can become risky in the context of substance use.

**Methods:**

In this ethnographic inquiry, 25 women with substance use disorders (SUDs) were included at two Drop-In-Centers (DICs) in South Tehran. Observation, semi-structured interviews and field notes were used to collect data. Qualitative content analysis was used to attain the explanatory model of women’s sexual behaviors in the context of substance use.

**Results:**

Three major themes emerged from the data analysis regarding their lives in the context of substance use; 1) life in the context of drug abuse, 2) negative self-perception, and 3) strive to survive. Subthemes were identified as loss of contact with family, social stigma, self-forgetfulness, worthlessness, low self-efficacy, and unsafe sexual context.

**Conclusion:**

Findings suggest that women with SUDs are highly interwoven with women’s sexual health, facilitating a shift towards risky behaviors. Integration of safe sexual skills building programs with substance use treatment is needed.

## Introduction

Substance use disorders can be hurtful to health (1). Constant substance use can be caused by several risk factors like lower socioeconomic position and reduced social support (2). These factors induce dependency that is sexually risky. According to theories, there are associations between high risk sexual activity and substance use (3). In Iran, the number of drug dependent women is unknown, but the number of women using stimulants is rising. A study conducted in Tehran reports 81% of female drug users consume crystal or methamphetamine (4). It is estimated that 9.7% of people with drug dependence are women who started drug use between 15 and 19 years of age. Among women (90%) who use methamphetamines, most are under the age of 35 years (5). Discussions of the relationship between drug abuse, gender and sexual risk behaviors have focused on the initiation of high risk sexual behaviors for women, sexually transmissible infections and domestic violence (6). According to Jessor and Jessor’s theory of “problematic behavior”, drug abuse can diminish one’s understanding of vulnerability and negatively impacts judgment power, safe behavior and the ability to make sound decisions (7). Substance use can make risky sexual behavior more likely to occur. Half of Iranian drug using women engage in risky sexual behaviors (8). Drug using women are often involved in commercial sex work (CSW) primarily as a result of lack of access to legal employment (9). Other predisposing factors in joining CSW industry are also pointed out such as: being single mother and living with a drug using partner. Female sex workers are often abused by their sexual partners, police, or by pimps, the people who provide them with a place to meet their clients (10–13). Limited power in negotiation for protective behaviors and safe sex acts increases the risk of sexually transmissible infections and HIV (14).

In Iran, like many countries, women’s addiction is highly stigmatized. Significant association between drug abuse and experience of domestic violence by sexual partners has been reported (15). Women are considered the core of family in child rearing, and in the context of drug addiction they will be exposed to severe social stigma, discrimination and exclusion (16). Most Iranian women who use drugs live in an inappropriate and unhealthy home situation and engage in sex work to support themselves, their children and sometimes their addicted husbands (17).

In 2015, 30,183 Iranians were living with HIV and 4,572 (15%) of these were women (18). Overall, 4.8% (2.2 to 9.8) of these women had a history of drug injection (19). It is estimated that 4.5% of HIV positive women are sex workers (20). A national wide study in Iran points out over 70% of female sex workers (FSW_s_) ever used drugs and 15% with injection history. According to this study, HIV prevalence among FSW-IDUs is 8.5% (21). The majority of sex workers report no condom use during their last sexual intercourse with a client (19, 22). It has been estimated that 22.1% of FSWs in Sajadi et al.’s survey never used condoms in any form of sexual contacts (19, 23). It is reported that they often have a low awareness about the adverse consequences of risky sexual behaviors (24). A study with Iranian drug using women reported 10% women who used drugs and were referred to MMT and were involved in unsafe sexual behavior (25).

With such a warning situation among Iranian women with risky behaviors, our main research question was developed to investigate how women’s sexual behavior can become risky in the context of substance use. Providing an insight into the personal experiences and perceptions of the women’s sexuality and drug dependence can be important in developing gender specific and culturally appropriate interventions in the field of addiction and sexual health.

## Methods

Our ethnographic study was done from January 2014 to February 2016. Ethnography demonstrates the formation of human experiences and how they give meaning and interpret their life experiences (26). Ethnography was employed because the main researchers (EMK & MJ) were involved with female drug users and sex workers for more than a decade. Harking back to Janice Morse’s viewpoint, the researchers’ formal and informal encounters would have authorized their role as an insider as well as outsider (27). However, in addition to ethnographical approach, content analysis was triangulated to reach an in depth insight into women’s perceptions toward risky sexual life. Using method triangulation, an attempt was made to actively be engaged in learning about how women’s sexual behavior can become risky in the context of substance use. A researcher could accept a culture by gradual cultural immersion and should try to increase his/her knowledge with language, cultural-social norms, traditions, verbal and non-verbal communication patterns, expression of emotions and feelings of people (28). In this study, 25 women were included at selected Drop in Centers (DICs) who were 18 years or older, who had a substance use disorder and were likely to have engaged in sex work. There are few DICs in Tehran, and most of these centers are located in southern Tehran due to the concentration of drug users and other risky population such as sex workers. In our initial assessment and context mapping, only four of these DICs were found appropriate to be selected as a research field. In this study, only two of four volunteered to participate. These centers had maximum variation in conditions of substance use women such as lifestyle of women, their previous living condition such as living in shelter, sleeping in cardboard, homelessness, and living at home with a sexual partner. Each of these centers had approximately 1,000 to 1,500 registered cases from these women. DICs provide health services including health education, maintenance therapies, and sexual health services. The objectives of the study were explained and verbal and written consent were obtained prior to data collection. Long-term attendance of the researchers (MJ and EMK) as midwife and sexual health specialists in these centers facilitated rapport, familiarity with the context, clients and recruitment. The participants networking facilitated access to other women outside the DIC who were called outreaches (Volunteer women who had history of substance use disorders); the women in DIC invited other women to attend our group sessions. Some of women volunteered to be interviewed individually. Women (n=19) attended in four focus group discussions (FGDs) with five participants in each group and seven women were interviewed individually (One from FGDs asked for individual interview). Individual interviews were also conducted with six women, who didn’t attend FGDs, because of their unavailability at the time of the FGDs. The purpose of the FGDs was to explore the women’s current drug problems related to their sexual life alteration. The interviews were conducted using interview guide (Appendix).

All interviews (60 to 90 *min*) were audio recorded and transcribed by the research team verbatim. All notes and audiotapes were coded using the principles of content analysis; substantive statements were identified and emerging patterns noted (29).

To open their layers of life, behavioral experiences and patterns, conventional content analysis was employed to focus on creation and development of categories and interpretation of writing with inductive method. Hidden contents and models can be clarified from the content (30, 31).

All the activities in different stages were performed to confirm the overall agreement on the substantive statements according to Speziale’s evaluation (29).

In this naturalistic inquiry, Lincoln and Guba approaches (1985) were employed to achieve our research rigor including prolonged engagement and persistent observation to establish the credibility of the study by methods triangulation, data source triangulation, investigator triangulation, external checks, participants debriefing and checking, searching for disconfirming evidences as well as researcher credibility as a valid instrument to interview women and collect field data (32).

### Credibility:

To ensure that the study describes the participants’ experiences, and the performed activities in all stages were recorded accurately (Repeated listening to interviews, coding, continuous comparisons, subthemes and themed extraction until the final themes), the interviews were read and the text was coded and a group met the participants to ask them to confirm the validity and proportionality of the extracted codes with their experiences and opinions.

### Transferability:

For the matter of transferability, interview with maximum variation was done; for this reason, participants aged 18 and above, and various level of education, social, sexual experiences, and people with different beliefs were included and even two substance dependent women who were not sex workers were also interviewed (These women had husband and lived with them in the home and the women asserted that they were not with other men).

### Dependability:

The stability of qualitative findings was ensured; for this reason, the finding was reviewed by two expert members of the research team who were experienced colleagues in qualitative studies.

### Conformability:

For this purpose, the whole process of data collection, analyzing and extracting the themes was explained fully so that others could read and distinguish the research.

Throughout the research, pseudonyms were used to protect the participants’ identities. The ethics committee of the Tehran University of Medical Sciences approved the study (Approval code 93-2-49-25273).

## Results

**Figure 1.**
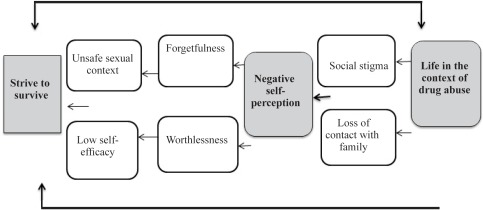
Sexual risk behavior in Iranian drug dependents women

Three substantive main themes were extracted as the main elements of the explanatory model of women’s sexual behaviors with substance use disorders. The themes included: 1) Life in the context of drug abuse; 2) Self-disturbed perception; 3) Strive to survive. Subthemes were identified as loss of contact with family, social stigma, self-forgetfulness, worthlessness, and low self-efficacy, and unsafe sexual context (Figure 1).

### Life in the context of drug abuse:

Drug abuse provided changes in the life of majority of the participants. The most important change in their sexual interactions was daily concern to obtain drugs. It caused “indifference”, “neglect of family life” as well as poor help seeking behaviors such as HIV testing, seeking for or using condom consistently. The majority of women defined these changes with the concept of “cutting off” from the family, and they considered the effects of drug abuse as the starting point of the mentioned experiences.

### Loss of contact with family:

A participant described herself such, under the effect of drug abuse and said “substance makes you careless; you do not have any plan for your life. You ignore your family or health. You concern is just for drug as if it ended. You are not thinking about going somewhere for testing like HIV or Hepatitis”. She was 39 years old, who used to smoke opium with her husband and has used opium for 19 years (FGD4).

A participant attributed her separation from her family and involvement in sex work to her addiction. “I was 16, I ran away from home because of my addiction. Maybe if I had not run away, I was not drawn this way, and I am a sex worker now, because I do not like this situation”. She was a 32 year old sex worker and had been using opium for 12 years (FGD2). As other participant said “my husband and I really loved each other at first, but problems gradually increased due to drug abuse. If someone in the family had asked what’s wrong with you, I would not have separated from my husband”. She was 36 years old with a 17 year history of drug abuse (FGD3).

Some Iranian families consider separation from husband a terrible outcome which in turn isolates women when their marriage breaks down. A participant said “I was 14 years old when my stepmother arranged my marriage to a man who was 28 years old. I never loved him. I got divorced. My family did not accept me and abandoned me. … I met another man who was a drug dealer. When he was in prison, I was addicted and homeless”. She was 38 years old, who had used drug for 14 years (FGD1).

Narratives of FGD1and FGD3 are similar to other participants. Divorce and addiction are two interrelated phenomena where either can cause the other. Divorce is a negative status in Iranian culture for women, and women are prone to social impairment after divorce. “Feeling humiliated”, “rubble” and “unprotected” were terms divorced women used to describe their situation.

### Social stigma:

The findings highlight the point that the stigma attached to drug use can lead women to lose their social and family supports. Some women choose to leave the family because there is no affection, intimacy or togetherness, and nobody cares or wants them as a member of the family. In describing the formation of risky sexual behavior, women with SUDs eventually realize that there is no opportunity to secure employment in accordance with society’s norms or to gain financial independence. They are unable to believe in their ability, capability and confidence to provide their basic needs or to achieve their life goals. These negative self-perceptions are created by stigma originating from society at large. Therefore, it seems that they have changed their view of their sexual potential and values, and internalized the stigma that they always would be exposed to sexual misuse or abuse. As a result, they resort to having sex with strangers in order to survive. A participant said “when you go to work, the employer first looks at your appearance, and when he sees your teeth, … then wants you to go with him, when you finished your work and others went home…umm… you have to stay….[to have sex with him]”. she was 35 years old and has been a drug user for 19 years (FGD4).

### Negative self-perception:

The narratives reveal that women themselves hardly accept “female addicts” and tried to isolate themselves from the community. They had internalized the reality that “a female addict deserves to be isolated or segregated”.

Like other women in this study, a participant thought women should be abandoned from the community. She said “Addicted women deserve to be isolated from the community. Other problems such as divorce are the results; she even humiliates herself and always step backs from the community. … well you have to step aside from your family, community”. She aged 29 and used drug for 9 years (FGD2).

Another participant supported other participants’ viewpoints. “After separating from my husband, I went to work, but I worked for 2 months and then was thrown out due to my addiction … I always wanted to quit drugs”. She was 44 years old and had used drugs for 22 years (FGD3).

### Self-forgetfulness:

Self-negation was found an important consequence of living in the context of drug use. Women perceived their addictive behaviors as what would ruin their lives. In other words, they used to refer to themselves as “dead body due to using drugs”.

Women’s narratives show they are used to disregarding their own welfare over others. Women believed that “an addict woman is a fallen woman and deserves to be used as a sexual object”.

“Worthless feminine” was a common language women employed to describe themselves. From the women’s viewpoint, a worthy woman is free to express her power, make a decision and have a life plan, while our participants found themselves hopeless about the future, uninterested, and incapable of making their own decision. Women point out their ruined femininity as the reason for separation and isolation from society. Further, the participants were deviated from their learned gender role. They had easily degraded their gender-based values as well as roles. They no longer saw themselves as ‘a woman valued by the society’ so that it didn’t matter if they were engaged in highly stigmatized and forbidden behavior such as sex work. In other words, these women have defined another construction for their gender role in the context of drug abuse. Many of the women felt damaged and worthless because they had been violated and abused physically, sexually and psychologically, as it is evident from their narratives. Regarding the above issue, a participant said “I do not have anything to lose in life, you know…I have totally forgotten myself [khod faramoshi]”, “I have lost the best times in my life”, and “I regret and want to return to the past”. She was 37 years old who has used drugs for 15 years (FGDs).

### Worthlessness:

Women who felt “worthless” never thought they would have a chance to enjoy themselves. The underlying impact of “self-negation” and “worthless feminine” on the women’s sexual lives was reflected in their narratives. Self-negation in this context specifically what could be called “self-sex-negation” is a by-product of drug abuse and selling sex. The drug dependent women’s viewpoint was that sex work impairs their existence. They turned to be a “fallen woman” or “disembodied feminine”.

One of the participants highlighted her feeling about selling sex and said “I’m not a woman, if I were a real women, it would be my right to enjoy my body…my sex… why (am I) experiencing sexual harassment?! Men should not have looked at me like a sex worker well I am addict ... that’s OK! …”. She was 32 years old who has used drugs for 12 years (FGD1).

“(A) friend of mine sells sex …(I) asked her why she was doing that when she knows about risk of that and AIDS and other stuff…she said when I am homeless … must live in a house or in the street … well I do not care whether I die a year earlier or live longer… (a) worthless one must die sooner … you know”. She was 44 years old who has used drugs for 16 years. She never said she was sex worker but talked about her girl friend who was an addict and a sex worker (FDG2).

It seems that the change in these women’s sexuality in the context of drugs may not be different from that of their Western counterparts. The differences causing the ambivalence in sexual discourse in Iran seemed to be evasions, negations of femininity, and ignorance of their sexual rights. Feeling worthless clearly put women in the context of risk as reflected from their narratives. They might have ignored using safe sex skills knowingly and emotionally in order to die sooner.

Strive to survive. Our findings showed that earning money was the primary reason for women to become sex worker. The majority pointed out the fact that a participant narrated “We have (a) right to save our lives, nothing left to sell…but our bodies”. She was 29 years (FGD3).

The majority of women referred to “saving their marriage” as another reason to be a sex worker. Many of them were forced by their husbands to support whole family financially as well as provide her husband’s and sometimes son’s drugs. “… what would you do? Divorce is far more dangerous than…[SW], my marriage was on the edge…you know…we [women] have to do anything to save our marriage…otherwise my kids become like their father…no matter what I am doing; I am a good mother instead…”. She was 49 years and a sex worker for more than a decade and rationalized her involvement in sex work (FGD2). From these women’s viewpoints, involvement in sex work is sometimes a way to save their family’s life and keep marital life safe and steady. It was a dominant belief among the participants.

As a participant said “selling body should not be a huge guilt in God’s sight if it was for saving one’s life”. She was 29 years (FGD3).

### Low self-efficacy:

The lack of self-confidence caused by social stigma was a common problem which adversely affected their efficacy in problem-solving. Women’s narratives supported the hypothesis that women tend to act as ‘self-destructive’ and hardly tend to change their unhealthy or risky behaviors. In other words, some of them consciously avoid using condom and put their lives at risk of STIs. One of the participants said “(It is) not easy to be sex worker…I was told condom protects people from getting sick…but if I become sick and die…the sooner the better…”. She was 38 years and a sex worker for 15 years (FGD3).

Two women who were sex workers were interviewed in our study, but they didn’t use any drugs. They told they even use condom for sex work with their partners.

### Unsafe sexual context:

The participants argued that they accept unsafe sexual behavior due to gender role alteration in the context of drug. From their point of view, women who use drugs are considered totally fallen and do not deserve to have a healthy and happy life unlike those women who are not labeled as an addict. These women strongly believed that “they are not an acceptable individual and worthy woman” as expected by the society.

Majority of women highlighted gender based subordination as an influencing factor in their submission to men’s power in the Iranian culture. For example, a woman has basically the intention to satisfy her partner’s sexual will (Such as refusing condom use) in order to keep her relationship safe with the partner. As a participant said “do you know what it means… sleeping in the street at night, well you have to stick to a man and give him a good service [sex]….then he can support you; … he can fight with a stranger who may want to rape you … even if he is homeless, I prefer to be with him”. She was 39 years old and a poly drug user (FGD3).

Women with substance dependency pointed out the main restraints of unsafe or risky sexual behaviors among women with drug problem such as ‘money for drug, unemployment, social rejection, stigma, and unpleasant sexual life’. In this line a participant said “I feel so bad… unpleasant …but no more option…[crying]… he gives me drugs, food and shelter at night … how can I ask him for condom? I know…I know he sleeps with many others …”. She was 27 years old and a crack user (FGD1).

Traditional gender role attitudes were identified by women’s narratives. They emphasized stereotypical norms about women with drug problem compared with their male counterparts in the Iranian culture. From the participants’ viewpoints “being a woman” was strongly associated with unsafe sexual behavior for substance using women.

## Discussion

In this interpretive approach, the shift in sexual behaviors from the norm to deviated and risky patterns was explored in the context of drug use for women in Iran. According to the narratives of women, “family support” seems to be an important factor in protecting women. If they have family support they won’t involve in risky sexual behavior. Some defined this as financial support, and others described it as psychosocial support. When women were affected by drugs, they described themselves as a neglected person who loses contact with the family and control over her social life as a careless individual. Even though her family did not reject her, she felt she had to leave her family to obtain drugs.

Turning sexual behaviors from healthy into risky patterns is common among drug users. The issue is mentioned in other studies like in South Africa where women with substance dependency are exposed to violence and a range of unsafe sex behaviors, including high levels of gender inequity and the disempowerment. This issue has effect on women’s ability to protect themselves. They couldn’t use condom with sex partners (33–36). Our participants had used substances prior to being a sex worker, whereas in one studythey had used stimulant and opiate following the beginning of sex work with men (37).

For our participants, risky sexual behaviors were associated with their lives in the context of SUDs by two major concepts, 1) internal feeling; being worthless, self-sex-negation, low self-efficacy and 2) external circumstances; stigmatization, divorce, inability to support themselves.

Labels and stigma related SUDs are social and cultural processes in many countries like Indonesia and Vietnam that may lead to unacceptability and unworthiness at individual, social and cultural levels (38, 39). Drug use in Iranian community like many other countries in the world has the high degree of stigma especially for women. It is a mental process with negative feelings about self that can be caused by personal experience resulting from negative social reactions. “Self-disturbed perception” has been shown as an influencing factor in risk taking behaviors, because the concept of self is the third important factor that affects development of personality after genetic and environmental factors. A person who understands the concept of self tends to perceive everything through the perspective of motivation, imagination and feeling (40). Our findings highlighted the risk-taking pattern when women narrate their sexual lives after becoming addicted. In fact, the social stigma is a cliché that leads to the formation of an underground group (39).

Other variables influencing the vicious cycle of “negative perception” of the women were identified and they included loss of family support, unemployment, lack of shelter and money. Living in this cycle led women to feel worthless and experience rejection.

Feeling of worthlessness appears to be strongly associated with high-risk behavior in these women. The person with poor feelings of self-worth and low self-efficacy may be principally vulnerable to drug use as a way of escaping from their negative feelings. The result of one study revealed that there was correlation between low self-worth and engagement in risky behaviors such as using higher levels of cigarette, marijuana, and other illegal drugs (41). Feeling worthless also shows that various reasons lead one to take risks such as overcoming the shame of sex work or taking drugs to relieve the stress caused by life’s emotions, poverty, family conflict, and divorce (42). Divorce related stigma is a gender-based phenomenon as it is different for men and women in the Iranian culture (43). Alteration of people’s norms determines specific behaviors which are expressed by them (44). Our findings suggest that drug dependence has adversely affected the women’s gender role scripts. In the Iranian culture, a woman is respected when she is pure which indicates her dignity and chastity throughout her womanhood. While a drug using woman is socially stigmatized, her dignity and honor would be fully destroyed. As a result, the woman would receive no support from her society mentally and economically. She, therefore, uses her sexual potential as a means to struggle for survival.

Negative perception was evident when women described their sexual understanding and sexual experiences. This is in line with the definition of Hilbert for sexual behaviors as “all actions and behaviors happen related to one’s gender role behavior or sexual behavior” (45). Our findings reveal that women’s life instinct has been diminished or disappeared, because they are used to seeing themselves as deserving to die and interpret an addicted woman as ‘worthless’. Therefore, they ignored living, enjoying, and being productive as Hilbert believes that the imbalance of power related to one’s gender role plays an important role in gender intermediaries, such as healthy or unhealthy relationships (45). Regarding self-forgetfulness and fallen woman or ineffective feminine, one can say females as sexual beings are depicted as sometimes ‘precious’ objects and at other times ‘harmful sexual beings’ who may put the honor of a family at risk; they are treated either sympathetically or strictly (46). Sexual relations in Iranian society and culture are defined concepts for couples; loyalty to husband seems to be an important component of a devoted woman and her sexuality (47).

Motivation for sex worker varies in different societies and cultures. In a number of countries, sex work is directly related to the tourism and immigration. Sex work is viewed as a business in different forms of having sex, nude paintings and movies, strip clubs, and pornography, phone sex or the internet. Sometimes, it is carried out to pay debts, to survive, or to simply earn a living (14, 48) while in our study sex work was constructed by some other concepts. They became CSWs to provide drugs for themselves and their spouse as well as shelter. In this study, interview could not be done with women who did not refer to DIC_s_ and this issue was one of our study’s limitations.

## Conclusion

The explanatory model drawn from the findings shows the close linkage between substance use disorders and sexuality of women. In the context of drug, women’s sexual behaviors indicated dramatic changes in risky sexual performance. Selling sex and unsafe sexual encounters were common for the following main reasons: women do not have comprehensive perception of risk due to being under drug influences; lack of enough knowledge and safe sex skills and alteration of their gender based sexual scripts. They were enforced to commit risky sexual behaviors and become CSW because they had to survive when they were divorced, unemployed, or if they lost their family support and became homeless.

Taken together, our findings reveal that woman’s risky sexual behaviors, defined as unprotected sex, sex work, and coercive sexual performance, are influenced by a constellation of gender-based norms consistent with theory of gender schema and sexual script. That is, gender norm appears to influence the impact of social norms toward a drug using woman. It also influences believing in an inability to have control over sexual behaviors in an unsafe context (*i.e*. drug use, sex working, and homelessness). Norms and sexuality of a drug user seem to have profound impact on condom use and intentions to engage in unsafe sex.

This study was conducted with those who were drug users and sex workers. In other words, there was no insight into the standpoints or experiences of sex workers who were not drug users. Adequate and culturally comprehensive assessment in Iranian women with SUDs is recommended. Gender specific programs are critical to improve treatment outcomes and sexual health for women who are drug users. The findings of this study can be used as a basis for interventional studies, and in the content of those interventions, themes were revealed which may help to prevent these women’s high risk sexual behaviors.

## Appendix

**Appendix.** Interview guide for semi-structured interviews

**No.**	**Questions**
**1**	What do you mean by a safe sex action?
**2**	What do you know about sexually transmitted infections?
**3**	How do you help your sexual health?
**4**	How do you protect yourself against HIV or Hepatitis B and HPV?
**5**	Why does a woman involve in unprotected sex?
**6**	Why do you choose this specific sexual relation?
**7**	How does a woman’s sexual life look like when she is involved in substance use?
**8**	What happens with your sexual behaviors/relationships when using drugs? (*e.g*. multi-partners, selling sex *etc*)?
